# Fair and robust early readmission risk prediction from electronic health records via diffusion-based data augmentation and causal-invariant representation learning

**DOI:** 10.1038/s41598-026-50146-5

**Published:** 2026-04-27

**Authors:** Haodong Lu, Jian Xu

**Affiliations:** Department of Information Management, Xinhua Hospital, School of Medicine, Shanghai Jiao Tong University, Shanghai, China

**Keywords:** Multiracial diabetes risk prediction, Medical fairness, Diffusion-based generative model, Causal-invariant representation, Domain generalization, Computational biology and bioinformatics, Health care, Mathematics and computing, Medical research

## Abstract

This study addresses the challenges of performance degradation and decision unfairness in multi-ethnic electronic health record data for early readmission risk prediction in diabetes, where racial distribution shifts and sample imbalance often lead to inconsistent generalisation across demographic groups. To tackle these issues, a unified framework is proposed that integrates the MedFair Diffusion Block with Causal-Invariant Domain Generalisation to jointly improve robustness and fairness in cross-racial prediction. The MedFair Diffusion Block is designed as a source-side, fairness-oriented augmentation module trained only on the training split of the designated source domain. It generates fairness-enhanced samples to alleviate source-domain imbalance and underrepresentation without accessing target-domain data, thereby avoiding information leakage during cross-domain evaluation. On this basis, the Causal-Invariant Domain Generalisation module maps both original and fairness-enhanced source samples into a shared latent space, disentangles relatively stable predictive factors from group-sensitive variations, and strengthens structural alignment through invariance constraints to improve cross-group transfer stability. Comprehensive experiments on the multi-ethnic Diabetes 130-US hospitals 1999–2008 dataset show that, compared with several representative baselines, the proposed model consistently improves predictive performance and fairness across four source-domain settings. In particular, it achieves average gains of approximately 2.0 percentage points in AUC and 1.5 percentage points in accuracy, while reducing Demographic Parity Difference and Equalised Odds Difference by more than 25% on average. These results indicate that the proposed framework provides a more balanced and stable trade-off between predictive performance and racial fairness under single-source cross-racial generalisation.

## Introduction

Diabetes has been rising rapidly worldwide, and risk prediction models based on Electronic Health Records (EHR) have become increasingly important tools for supporting clinical decision-making and resource allocation^1^. A large body of literature has shown that modeling structured visit information can effectively assist in predicting readmission risk, the progression of complications, and long-term outcomes, thereby enabling earlier intervention and more precise management^2^. However, substantial disparities exist across patient groups with respect to race, socioeconomic status, and healthcare-seeking behavior. When models are trained solely on a single population, they often exhibit performance gaps and decision inconsistencies across racial subgroups under distributional shifts, which may further exacerbate existing health inequities^3,4^. In multi-ethnic EHR settings, these challenges are further amplified by several intertwined factors. First, different racial and ethnic groups often exhibit substantial heterogeneity in disease burden, access to care, treatment trajectories, and follow-up behavior, leading to pronounced shifts in both feature distributions and outcome patterns across groups^5,6^. Second, minority groups are frequently underrepresented in real-world clinical datasets, causing learned models to be dominated by the statistical regularities of majority groups and making prediction errors more likely to accumulate in underrepresented populations^7^. Third, historical inequities and imperfect clinical documentation may introduce latent structural bias into EHR systems, such that models trained naively on observational data may inherit and even amplify pre-existing disparities^8,9^. In addition, medical prediction settings are particularly vulnerable to dataset shift, in which changes in population composition, documentation style, and care processes may substantially weaken both generalization and fairness during deployment^10^. Consequently, achieving both high predictive accuracy and group-level fairness under multi-ethnic heterogeneous data conditions has become a critical challenge for next-generation clinical intelligence systems. To better contextualize these challenges in multi-ethnic EHR-based early readmission risk prediction for diabetes, Table 1 summarizes the key sources of distributional disparity and fairness risks highlighted in prior studies.

**Table 1:** Overview of key challenges in multi-ethnic EHR-based risk prediction.

Challenge Category	Brief Description
Population distribution disparity	Different racial and ethnic groups exhibit substantial differences in feature distributions, disease exposure, and healthcare utilization patterns, leading to performance degradation when models are transferred across groups.
Sample imbalance	The limited number of samples from minority groups makes the model more likely to favor statistical patterns of majority populations, resulting in biased predictions.
Structural bias	Structural disparities in healthcare behavior, access to medical resources, and social determinants may be amplified by the model, giving rise to implicit unfairness.
Insufficient domain generalization	Existing methods struggle to maintain both accuracy and stability on heterogeneous data, and their cross-domain performance is generally limited.

Existing approaches tend to focus either on improving overall accuracy while overlooking cross-racial generalization and fairness, or on enforcing simple fairness constraints that reduce group disparities at the cost of robustness in out-of-distribution scenarios. Although recent studies have emphasized fairness auditing, bias diagnosis, and robustness under distribution shift, most existing methods still treat fairness enhancement and domain generalization as separate objectives, rather than jointly modeling their interaction within a unified clinical prediction framework^11–13^. Moreover, generative modeling has recently shown promise for improving fairness under medical distribution shifts, suggesting that source-side data enhancement may provide a practical path toward reducing imbalance-induced bias while preserving predictive utility^14^. Moreover, most domain generalization methods fail to incorporate medical priors or structured clinical information, making them insufficient for characterizing minority subgroups with limited samples. To address these limitations, this study proposes a unified causal–fairness modeling framework for multi-racial diabetes-related early readmission risk prediction. First, this study develops the MedFair Diffusion Block (MDB), which is defined as a source-side fairness-oriented augmentation module trained only on the training split of the designated source domain. Instead of modeling cross-group empirical mixtures, MDB generates fairness-enhanced samples from source-domain data to alleviate source-side imbalance and underrepresentation without accessing target-domain data, thereby reducing the risk of information leakage during cross-racial evaluation. Second, this study introduces the Causal-Invariant Domain Generalization (CIDG) module, which decomposes and extracts relatively stable causal factors in the latent space and explicitly aligns structural representations between source-domain samples and fairness-enhanced samples, thereby improving predictive stability and decision fairness during cross-racial transfer.

The main contributions of this work are summarized as follows:

1. This research propose a fairness-aware diffusion enhancement framework, MDB, which constructs a more representative and balanced source-domain training set through source-side fairness-oriented sample generation without requiring additional annotations or external data.
2. This research design the CIDG causal-invariant domain generalization module, which explicitly models stable semantics, cross-group variant factors, and fairness-related components within a unified latent space and employs invariance constraints to align the structural representations of source-domain and fairness-enhanced samples, fundamentally mitigating decision instability caused by racial distributional shifts.
3. This research establish a cross-racial source–target evaluation protocol on the publicly available Diabetes 130-US hospitals (1999–2008) dataset, and conduct comprehensive comparisons against representative baselines using Accuracy, AUC, Demographic Parity Difference, and Equalized Odds Difference. The experimental and ablation results jointly demonstrate that the proposed framework achieves significant and stable improvements in both predictive performance and racial fairness.

## Related work

### Fairness-aware medical prediction and bias mitigation across demographic groups

Recent years have witnessed growing attention to fairness issues in medical artificial intelligence, as systematic biases in model predictions across racial, gender, and socioeconomic groups are increasingly documented in real clinical environments. Huang et al^15^. provided one of the earliest structured examinations of the sources of racial bias in clinical machine learning models and highlighted that data imbalance is an inherent structural problem across many medical tasks. From a broader methodological perspective, Chen et al^16^. argued that fairness constraints should be incorporated simultaneously at the levels of model architecture, training objectives, and loss design. Chin et al^17^. emphasized the role of policy-level auditing in mitigating disparities, while the review by Cary et al^7^. further revealed that most clinical algorithms lack independent evaluation for vulnerable populations, leading to unstable fairness metrics in real-world deployment. In addition, Colacci et al^10^. noted that many biases do not arise from model design alone but rather from intertwined sociodemographic factors and clinical sampling processes. Building on these insights, Liu et al^18^. proposed an interpretable fairness modeling framework that helps uncover how models produce divergent decisions across demographic groups, and Hasanzadeh et al^19^. discussed a more systematic pipeline for bias identification and mitigation in broader healthcare AI applications. More recently, Rajkomar et al^20^. emphasized that fairness in clinical machine learning should be treated as a core requirement for advancing health equity, while Wang et al^12^. further showed that bias auditing is particularly important in readmission-related predictive models. Despite these advances, most existing methods depend on auxiliary demographic information or resampling strategies, limiting their applicability in resource-constrained and label-scarce clinical scenarios. Compared with these studies, the proposed method does not focus only on fairness auditing or post hoc bias analysis, but instead incorporates fairness-oriented sample enhancement and invariant representation learning into a unified predictive framework.

In terms of performance evaluation and fairness reporting for clinical prediction models, Chakradeo et al^21^. pointed out that current medical prediction studies lack adequate reporting of fairness metrics, making it difficult to reproduce or compare model bias across contexts. The systematic review by Siddique et al^22^. further demonstrated that performance disparities across racial groups remain a long-standing structural issue, affecting not only accuracy but also downstream clinical processes such as resource allocation and treatment recommendation. Naderalvojoud et al^23^. showed through opioid long-term use prediction tasks that latent biases in data can be amplified during model inference, ultimately shaping clinical management and medication strategies. Likewise, the assessment by Rountree et al^24^. revealed that most precision-medicine risk prediction models fail to systematically report fairness indicators, resulting in insufficient auditability and transparency. Overall, prior studies have identified substantial challenges related to racial fairness in medical AI, yet current approaches still lack systematic modeling of cross-group distribution shifts and overlook how semantic divergence and structural heterogeneity across domains influence model stability. Consequently, there is a pressing need to integrate fairness-aware modeling with domain generalization techniques to ensure robust and equitable performance across demographically diverse populations, thereby laying a foundation for more trustworthy and clinically deployable medical prediction systems.

### Domain generalization for robust clinical risk prediction across population shifts

As the deployment of clinical prediction models in real-world settings continues to expand, distribution shifts across hospitals, time periods, and demographic groups have emerged as critical challenges to model robustness. Guo et al^25^. systematically evaluated multiple clinical tasks and demonstrated that traditional models experience substantial performance degradation under temporal distribution shifts, underscoring the importance of domain generalization in healthcare applications. Zhang et al^26^. proposed transfer learning strategies to mitigate temporal drift and showed that long-term model deployment leads to significant performance decay. Extending this line of work, Guo et al^27^. illustrated that foundation models trained on EHR data exhibit stronger robustness under temporal variation, while Lee et al^28^. introduced a stable risk prediction framework that maintains consistent performance amid changes in electronic health record distributions. Hai et al^29^. validated the effectiveness of domain generalization techniques for cross-hospital and cross-population prediction in diabetic readmission tasks, revealing systematic shifts between patient groups from different sources. In multi-site investigations, Cabanillas Silva et al^30^. found pronounced model drift across medical institutions, highlighting the greater complexity of real-world distribution heterogeneity compared to controlled experimental environments. Complementing these efforts, Tosaki et al^31^. developed an OOD rejection mechanism for early disease prediction to enhance model safety under extreme distribution shifts. In addition, Schrouff et al^11^. showed that fairness transfer may fail under real-world medical distribution shifts, and Dockès et al^13^. further emphasized that dataset shift can substantially undermine the reliability of machine-learning biomarkers. Nevertheless, existing approaches often target a single type of shift–such as temporal or institutional variation–and have not fully addressed the compound nature of clinical domain shifts involving demographic structure differences, disease progression variability, and heterogeneous feature expression. Different from these methods, the proposed model specifically focuses on cross-racial clinical prediction and jointly addresses source-side imbalance and representation-level invariance under demographic distribution shifts.

In the areas of shift detection and cross-domain adaptation, the systematic review by dos Santos Silva et al^32^. summarized common shift patterns and mitigation strategies in medical prediction tasks, emphasizing that most methods lack the ability to jointly model multiple sources of distribution change. From an operational deployment perspective, Subasri et al^33^. proposed actionable protocols for shift detection and compensation, emphasizing the need for continuous monitoring and dynamic correction in real-world clinical AI systems. Koch et al^34^. demonstrated that models can rapidly fail post-deployment due to demographic structural changes, stressing the regulatory importance of distribution shift surveillance. Li et al^35^. introduced transport distance learning into medical fairness assessment, enabling consistent predictions across treatment groups, while Guo et al^36^. validated through multi-center studies that shared medical foundation models possess stronger adaptability in cross-institutional transfer. Moreover, Ktena et al^14^. recently demonstrated that generative modeling can improve fairness under medical distribution shifts, which provides additional support for the motivation of introducing a diffusion-based fairness enhancement module in the present study. Overall, despite substantial progress in shift detection, cross-domain adaptation, and model robustness, current methods remain limited in their ability to handle the compounded challenges posed by simultaneous source distribution change, institutional heterogeneity, and demographic structural shifts.

## Method

### Datasets and analysis

This study uses the publicly available *Diabetes 130-US hospitals for years 1999–2008* dataset from the UCI Machine Learning Repository. The dataset contains inpatient electronic health records collected from 130 hospitals and integrated healthcare facilities in the United States between 1999 and 2008, covering demographic attributes, diagnosis information, treatment records, laboratory examinations, medication usage, and hospitalization outcomes. Since all records correspond to hospitalized patients with diabetes, this study formulates the task as a *diabetes-related risk prediction* problem under a binary classification setting. Specifically, label 1 denotes patients who are readmitted within 30 days after discharge, and label 0 denotes patients without such early readmission. After removing samples with missing race annotations or invalid outcome labels, the remaining records are used to construct racial-group subsets for fairness analysis and cross-group generalization evaluation. The structured EHR features provide a suitable basis for studying predictive performance, medical fairness, and distribution shifts across demographic groups.

To evaluate group-wise distribution differences and their influence on model robustness, four racial subgroups are considered: Asian, African American, Caucasian, and Hispanic. Each record is assigned to one of these four groups and retains the same binary diabetes-related risk label definition across all experiments. Table 2 reports the sample size and preprocessing strategy for each subgroup. A substantial imbalance exists across groups, with Caucasian samples dominating the dataset, whereas Asian and Hispanic subsets are much smaller. To ensure comparability across source–target settings, a unified preprocessing pipeline is adopted. Missing values in continuous variables are imputed with medians, categorical variables with modes, extreme outliers are removed through quantile-based trimming, categorical features are encoded with unified mappings, and numerical variables are standardized to maintain a consistent feature space across all demographic groups.

**Table 2:** Sample counts and preprocessing rules for each racial group.

Race Group	Count	Missing Handling	Preprocessing Details
Caucasian	76099	Median/Mode Imputation	Quantile-based outlier removal; unified categorical encoding; numerical feature standardization
African American	19210	Median/Mode Imputation	Same preprocessing pipeline as Caucasian to ensure a consistent feature space across domains
Hispanic	2037	Median/Mode Imputation	Same preprocessing pipeline under a small-sample setting to reduce additional distributional inconsistencies
Asian	641	Median/Mode Imputation	Same preprocessing pipeline applied to preserve cross-group feature compatibility in domain generalization experiments

### Problem definition

This study addresses the fairness and generalization challenges of diabetes-related risk prediction under a single-source cross-racial domain generalization setting. Let the set of demographic groups be denoted by $\mathcal {G}=\{\text {Asian}, \text {AfricanAmerican}, \text {Caucasian}, \text {Hispanic}\}$. In each experimental setting, one group $s \in \mathcal {G}$ is selected as the designated source domain, and the remaining groups $\mathcal {G}\setminus \{s\}$ are treated as unseen target domains for out-of-domain evaluation. The source-domain dataset is defined as $\mathcal {D}_s=\{(x_i^{(s)}, y_i^{(s)})\}_{i=1}^{n_s}$, where $x_i^{(s)} \in \mathbb {R}^d$ denotes a *d*-dimensional clinical feature vector and $y_i^{(s)} \in \{0,1\}$ denotes a binary diabetes-related clinical outcome. To avoid ambiguity in the experimental protocol, all preprocessing operations that require data-dependent statistics, including missing-value imputation, outlier handling, categorical encoding, and numerical standardization, are fitted only on the training split of the designated source domain and then applied consistently to the corresponding validation split and all unseen target domains. Thus, the term “unified preprocessing pipeline” refers to the use of the same preprocessing procedure and feature space across all source–target settings, rather than estimating preprocessing statistics separately for each demographic group. Because different demographic groups exhibit substantial differences in both feature distributions $P_g(X)$ and conditional distributions $P_g(Y\mid X)$, a model trained only on $\mathcal {D}_s$ may experience performance degradation and group-level unfairness when directly transferred to another unseen target domain $\mathcal {D}_t$ with $t \ne s$. The objective is therefore to learn a prediction model $f_{\theta }:\mathbb {R}^d \rightarrow [0,1]$ using only the labeled data from the designated source domain, such that the learned predictor maintains robust predictive performance and consistent decision behavior on unseen target demographic domains without accessing their labels or distributional information during training, even under cross-group shifts satisfying $P_s(X)\ne P_t(X)$ and potentially $P_s(Y\mid X)\ne P_t(Y\mid X)$.

### Overall model architecture

The overall framework takes source-domain clinical samples as input and constructs a discriminative prediction backbone composed of an Encoder and a Causal-Invariant Domain Generalization Head, while introducing the MedFair Diffusion Block as a parallel sample-generation module under fairness constraints. The design motivation of the framework is to address two coupled challenges in cross-racial diabetes-related risk prediction: insufficient representation of underrepresented patterns in the source domain at the data level and unstable decision boundaries under cross-group distribution shifts at the representation level. Accordingly, the MedFair Diffusion Block is introduced to alleviate source-domain imbalance by generating fairness-enhanced samples from the training split of the designated source domain, whereas the Causal-Invariant Domain Generalization Head is used to extract stable decision-related semantics that remain less sensitive to demographic distribution changes. In this way, the former mainly improves source-side data coverage and structural diversity, while the latter improves cross-domain robustness and prediction consistency. Given a single preprocessed inpatient record $x \in \mathbb {R}^d$, latent representations are first extracted through operators such as multi-head self-attention, pointwise convolution, and normalization. The domain generalization head then aggregates robust predictive features and outputs the predicted probability vector $\hat{y}$ for the diabetes-related outcome. This backbone prediction mapping can be abstracted as

1 $$\begin{aligned} \hat{y} = f_{\theta }(x) = \textrm{DGHead}\!\big (\textrm{Encoder}(x)\big ) \end{aligned}$$

where $\textrm{Encoder}(\cdot )$ denotes the transformation integrating attention mechanisms and channel reconstruction, $\textrm{DGHead}(\cdot )$ is responsible for learning domain-invariant and causally stable decision patterns for cross-domain transfer in a shared latent space, and $\theta$ represents the learnable parameters of the discriminative backbone.

**Fig. 1:**
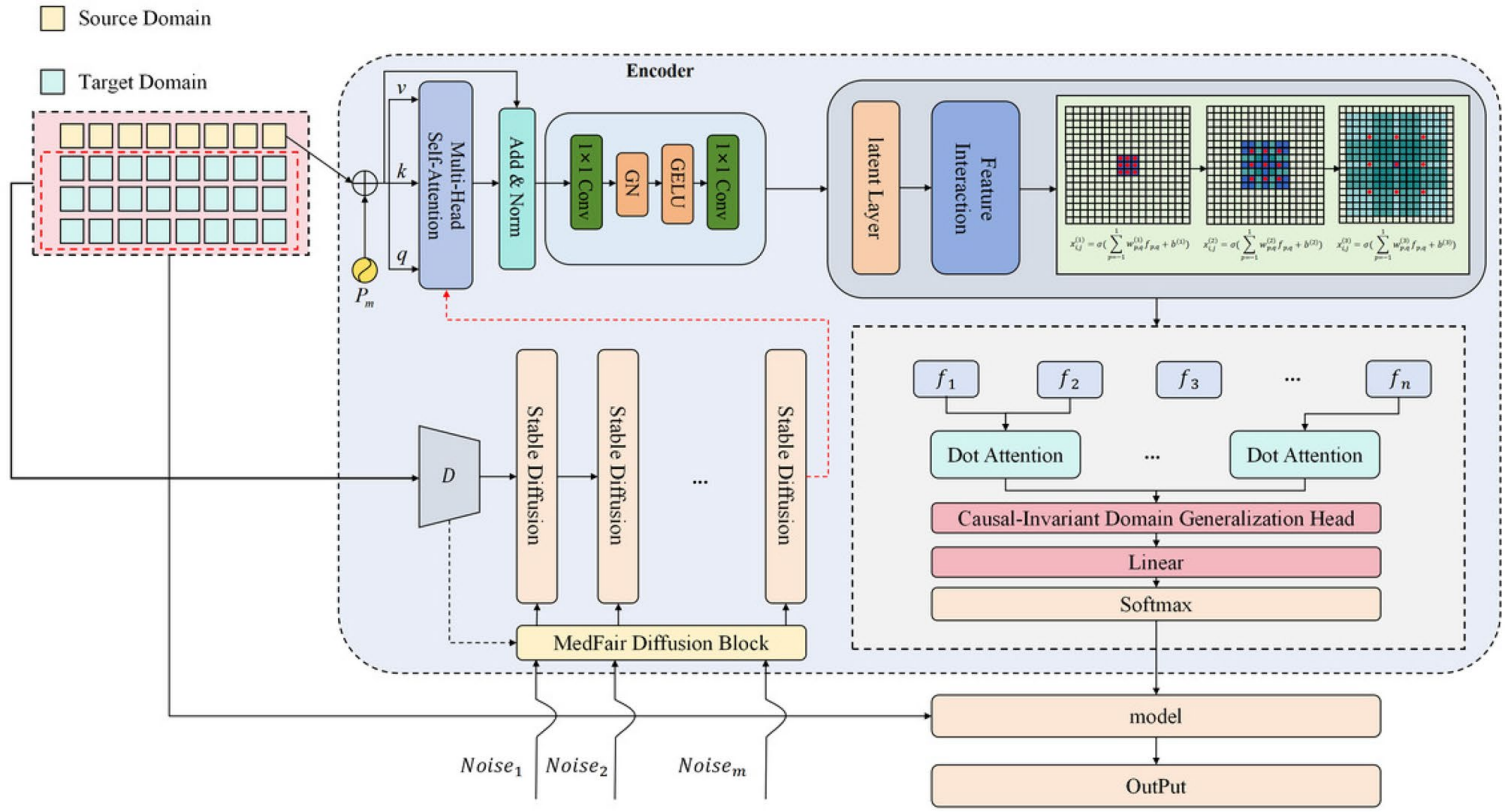
The encoder first maps source-domain clinical records into a shared latent space, capturing stable semantic representations through self-attention, convolution, and feature interaction. The MedFair Diffusion Block then generates fairness-aware augmented samples from the source-domain training data, while the causal-invariant domain generalization head aggregates multi-branch features and produces robust predictions for diabetes risk on unseen target domains.

Building upon this structure, the MedFair Diffusion Block is designed as a fairness-oriented generative module that synthesizes source-side fairness-enhanced samples. The module is first trained only on the training split of the designated source domain and is then kept fixed during the subsequent discriminative learning stage. Its role is not to directly participate in the final discriminative inference, but to provide a more balanced and structurally diverse auxiliary sample space for the downstream predictor. Given an original sample *x*, a conditional guidance variable $c_g$, and a noise vector *z*, the module produces a fairness-enhanced synthetic sample $\tilde{x}$ through a stable diffusion process, which can be formalized as

2 $$\begin{aligned} \tilde{x} = G_{\text {fair}}(x,z,c_g) = \textrm{MedFairDiffusion}(x,z,c_g) \end{aligned}$$

where $G_{\text {fair}}(\cdot )$ denotes the diffusion-based generative operator conditioned on medical priors and source-side fairness constraints. Here, $c_g$ denotes a unified conditional guidance variable used to specify the intended source-side balancing objective and medical prior constraint during generation. It may encode demographic guidance, fairness-control signals, or other structured conditional information, but it is introduced only as a high-level controller for fairness-aware sample construction rather than as a carrier of rich natural-language semantics. This formulation keeps the conditional generation framework sufficiently general while avoiding dependence on target-domain information during training. The prediction backbone can structurally accept both the original sample *x* and the synthesized sample $\tilde{x}$, thereby enriching the source-domain training distribution and providing a more balanced representational basis for subsequent fairness evaluation and cross-domain consistency analysis. Compared with relying only on the original imbalanced source data, this joint design improves source-side representation quality before causal-invariant feature learning is performed. The overall model architecture is shown in Fig. 1.

### MedFair diffusion block

**Fig. 2:**
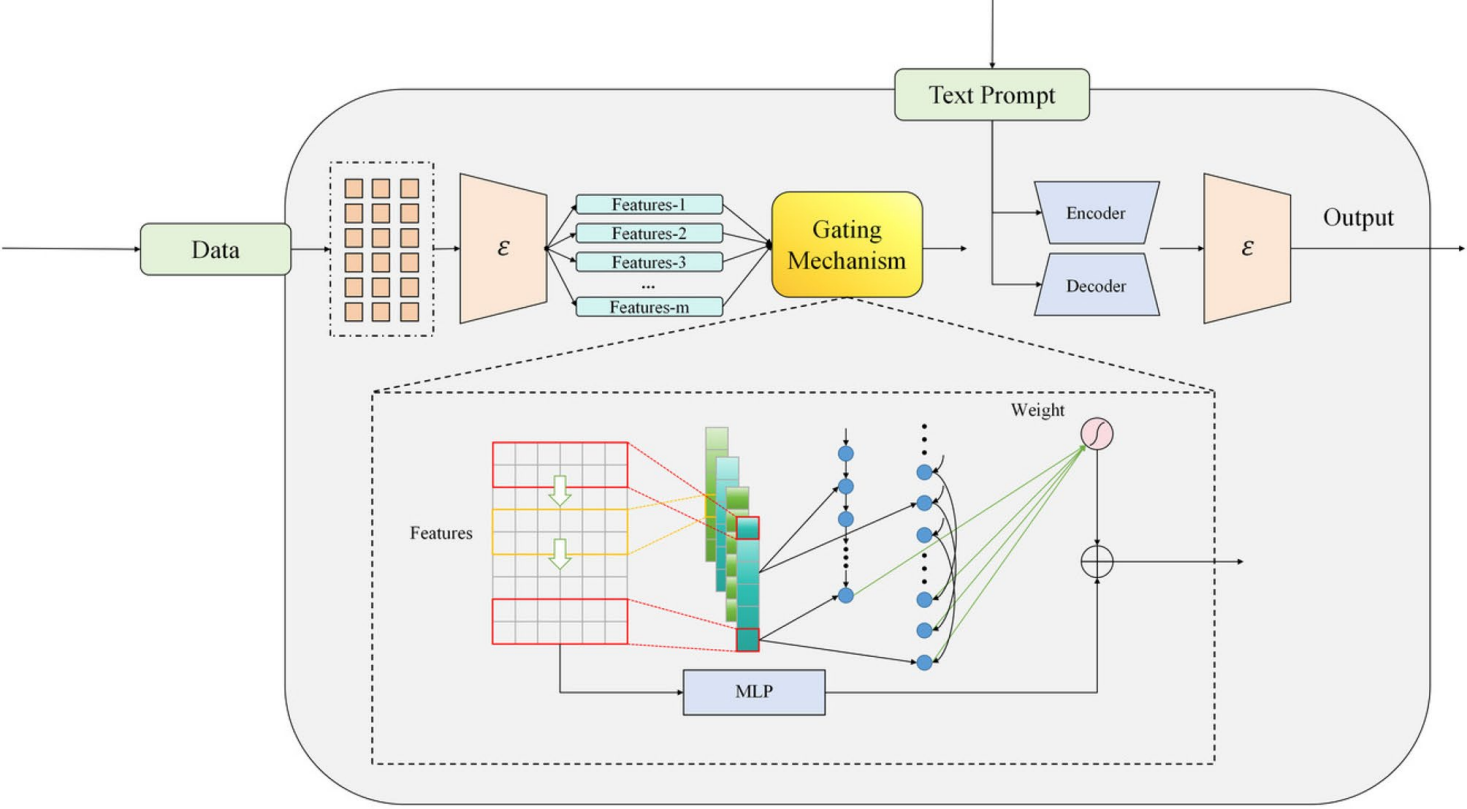
Architecture of the MedFair Diffusion Block, which extracts multi-scale features from source-domain clinical data, applies a gating mechanism guided by text prompts, and generates fairness-aware synthetic samples to enhance the source-side training distribution.

MedFair Diffusion Block is designed as an independent fairness-aware diffusion-based generative module, trained only on the training split of the designated source domain to construct a fairness-enhanced source-side distribution within the latent space. Its purpose is to alleviate source-domain imbalance and structural bias at the data level, so that the downstream prediction model can be trained on a more balanced and structurally enriched sample space. The overall architectural design is illustrated in Fig. 2.

Let the source-domain training dataset be defined as

3 $$\begin{aligned} \mathcal {X}_s=\{x_i\}_{i=1}^{N_s}, \quad x_i \in \mathbb {R}^d, \end{aligned}$$

where $x_i$ denotes the feature vector of the *i*-th hospitalization record in the designated source domain. To reduce the dominance of overrepresented patterns during the generation stage, the module constructs a source-side fairness-enhanced distribution:

4 $$\begin{aligned} p_{\text {fair}}^{(s)}(x), \end{aligned}$$

where $p_{\text {fair}}^{(s)}(x)$ denotes a fairness-oriented augmentation distribution estimated only from the source-domain training data. This design is adopted as a source-side rebalancing strategy rather than as an estimate of the real multi-group population distribution. Its purpose is to reduce the dominance of overrepresented source-side patterns and to improve the representation of underrepresented clinical structures during sample construction. Therefore, the goal of the MedFair Diffusion Block is to sample synthetic instances from $p_{\text {fair}}^{(s)}(x)$ through the diffusion process, thereby providing the downstream model with a more balanced and structurally enriched source-domain training distribution.

In the diffusion modeling component, the module adopts the standard forward Gaussian diffusion process, which progressively corrupts real samples with noise. For diffusion steps $t=1,\dots ,T$ with noise schedule $\{\beta _t\}_{t=1}^{T}$, the forward Markov process is given by

5 $$\begin{aligned} q(x_t \mid x_{t-1}) = \mathcal {N}\big (\sqrt{1-\beta _t}\,x_{t-1},\,\beta _t \textbf{I}\big ), \end{aligned}$$

where $x_t$ is the intermediate state at step *t*, and $\textbf{I}$ is the identity matrix. By recursion, a closed-form expression from the original sample $x_0$ to an arbitrary step *t* can be obtained:

6 $$\begin{aligned} x_t = \sqrt{\bar{\alpha }_t}\,x_0 + \sqrt{1-\bar{\alpha }_t}\,\varepsilon ,\quad \bar{\alpha }_t = \prod _{s=1}^{t}(1-\beta _s),\; \varepsilon \sim \mathcal {N}(0,\textbf{I}), \end{aligned}$$

where $\bar{\alpha }_t$ denotes the cumulative preservation coefficient and $\varepsilon$ is standard Gaussian noise. At step *T*, the process maps real samples to an approximately isotropic Gaussian distribution, forming a unified starting point for conditional generation.

To explicitly inject medical semantics and fairness constraints, the module receives a textual prompt *s* describing the task and fairness requirements, and encodes it using a pretrained text encoder $\textrm{Enc}_{\text {text}}$:

7 $$\begin{aligned} c = \textrm{Enc}_{\text {text}}(s), \quad c \in \mathbb {R}^{d_c}, \end{aligned}$$

where *c* serves as a high-level conditional signal for fairness-oriented generation. In the present task, the textual prompt does not aim to introduce complex natural-language semantics into structured EHR data, but to provide a unified control variable that specifies the intended medical generation objective and group-balancing constraint. Such a design allows the diffusion process to remain condition-aware while preserving a flexible interface for incorporating fairness-related guidance. Meanwhile, for any intermediate state $x_t$, the feature extractor $\varepsilon _{\phi }$ produces a multi-scale feature set

8 $$\begin{aligned} \mathcal {F}_t = \{f_t^{(1)},f_t^{(2)},\dots ,f_t^{(m)}\} = \varepsilon _{\phi }(x_t), \quad f_t^{(j)} \in \mathbb {R}^{d_j}, \end{aligned}$$

where $\varepsilon _{\phi }(\cdot )$ is composed of convolutional blocks and multilayer perceptrons (MLPs), *m* denotes the number of multi-scale or multi-branch feature streams, and $\mathcal {F}_t$ captures structural information across different local regions and semantic levels.

Based on these multi-scale features, the MedFair Diffusion Block employs a gating mechanism to adaptively select the feature channels most relevant to fairness. Specifically, each branch feature $f_t^{(j)}$ is concatenated with the text condition *c* and passed through a gating network $g_{\psi }$ to obtain its channel weight:

9 $$\begin{aligned} \alpha _t^{(j)} = \frac{\exp \big (g_{\psi }([f_t^{(j)},c])\big )}{\sum _{k=1}^{m} \exp \big (g_{\psi }([f_t^{(k)},c])\big )}, \quad j=1,\dots ,m, \end{aligned}$$

where $[\cdot ,\cdot ]$ denotes concatenation, $g_{\psi }$ is an MLP-based gating network, and $\alpha _t^{(j)}$ is the normalized importance coefficient for branch *j* at step *t*. This mechanism enables the model to assign different importance to different feature branches under the fairness condition, thereby emphasizing feature components that are more relevant to minority-group compensation and cross-group structural balancing. Using these coefficients, the multi-scale features are aggregated into a global condition vector:

10 $$\begin{aligned} r_t = \sum _{j=1}^{m} \alpha _t^{(j)} f_t^{(j)}, \quad r_t \in \mathbb {R}^{d_r}, \end{aligned}$$

where $r_t$ guides the reverse denoising process to emphasize feature structures associated with underrepresented groups.

In the reverse generation stage, the MedFair Diffusion Block initializes from isotropic Gaussian noise $x_T \sim \mathcal {N}(0,\textbf{I})$ and iteratively performs denoising using the condition vectors $r_t$ and the text condition *c*. Each reverse update is formulated as

11 $$\begin{aligned} x_{t-1} = \mu _{\theta }(x_t,r_t,c) + \sigma _t z,\quad z \sim \mathcal {N}(0,\textbf{I}), \end{aligned}$$

where $\mu _{\theta }(\cdot )$ is a denoising prediction function implemented using a conditional U-Net or Transformer, $\sigma _t$ is the step-dependent noise scale, and *z* is newly sampled Gaussian noise. Iterating from *T* down to 0 yields the generated sample $x_0^{\text {fair}}$, which approximates the fair distribution $p_{\text {fair}}(x)$. The final set of fairness-enhanced samples is denoted as

12 $$\begin{aligned} \tilde{\mathcal {X}}_{\text {fair}}=\{x_{0,i}^{\text {fair}}\}_{i=1}^{M}, \end{aligned}$$

where *M* is the number of generated samples. This set is used as an additional balanced data source during the training of the main prediction model, while the parameters of the diffusion module remain frozen throughout the discriminative training process, serving solely for fairness-oriented sample generation and distribution compensation without directly interfering with the primary learning objective.

### Causal-invariant domain generalization block

**Fig. 3:**
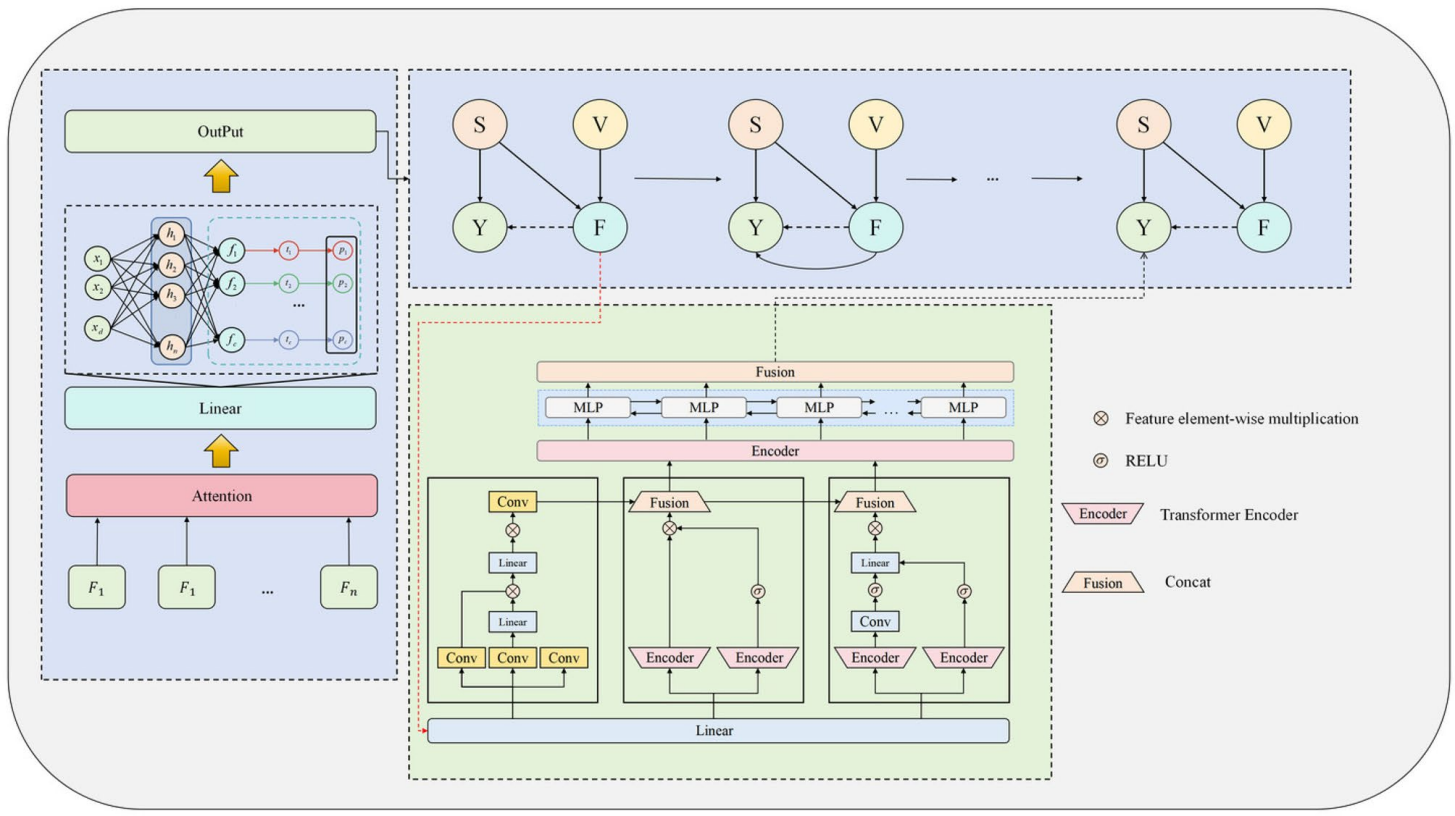
Causal-Invariant Domain Generalization Block integrates real source-domain samples and fairness-enhanced samples into a unified causal representation space by disentangling stable factors, variable confounders, labels, and invariant features. Through multi-branch fusion and Transformer-based structural modeling, the block extracts domain-agnostic causal features that support robust cross-racial clinical risk prediction.

The core idea of the Causal-Invariant Domain Generalization Block is to jointly map real source-domain samples and fairness-enhanced samples generated by the MedFair Diffusion Block into a causally invariant latent space, and to achieve robust prediction under cross-group distribution shifts through a structured causal representation learning mechanism. The objective of this module is not to identify fully observable causal variables from clinical records in a strict intervention-based sense, but to learn a set of latent components with different functional roles, so that stable predictive factors can be separated as much as possible from race-sensitive and domain-variant factors. The overall architecture of this module is illustrated in Fig. 3.

Let the source-domain data be denoted as $\mathcal {X}_{s}=\{x_i^{(s)}\}_{i=1}^{N_s}$ and the generated fairness-enhanced data as $\tilde{\mathcal {X}}_{\textrm{fair}}=\{\tilde{x}_j\}_{j=1}^{M}$. To integrate the two datasets within a unified space, both are first passed through a shared encoder $E_{\theta }$ to obtain the corresponding latent features:

13 $$\begin{aligned} h_i^{(s)} = E_{\theta }\!\left( x_i^{(s)}\right) , \qquad \tilde{h}_j = E_{\theta }\!\left( \tilde{x}_j\right) , \end{aligned}$$

where $h_i^{(s)}$ and $\tilde{h}_j$ represent the latent representations of source-domain samples and fairness-enhanced samples, respectively, serving as the foundation for building the subsequent causal-invariant structure. A shared encoder is adopted to ensure that both real and fairness-enhanced samples are projected into the same feature space, which makes subsequent invariant learning comparable across domains and prevents the representation gap from being dominated by encoder-specific discrepancies.

In the causal modeling stage, the module explicitly decomposes semantic factors (S), variant confounding factors (V), label variables (Y), and the final causal-invariant factors (F). To achieve this, a basic causal representation is constructed by mapping each latent representation into four structural variables:

14 $$\begin{aligned} S_i = f_{S}(h_i),\qquad V_i = f_{V}(h_i), \qquad Y_i = f_{Y}(h_i), \qquad F_i = f_{F}(h_i). \end{aligned}$$

Here, $f_{S}$, $f_{V}$, $f_{Y}$, and $f_{F}$ are multilayer-perceptron (MLP)–based mapping operators that simulate the four key variables in a structural causal model. $S_i$ captures stable semantics that are independent of racial groups, $V_i$ encodes the confounding factors with the largest cross-group variability, and $F_i$ represents the causal features used for prediction that should remain invariant across distribution shifts. In practice, these variables are not directly observed or manually annotated; instead, they are learned as latent projections of the shared representation through four parallel MLP heads with independent parameters. Among them, $S_i$ is intended to preserve disease-related semantic patterns that remain relatively stable across demographic groups, $V_i$ captures variation associated with group-dependent distribution shifts, healthcare utilization differences, and other latent demographic correlations, $Y_i$ provides label-oriented supervisory information, and $F_i$ is optimized as the final invariant feature branch used for downstream prediction. This decomposition serves as a functional representation split, allowing the model to organize heterogeneous information into relatively stable, variant, and prediction-related components within a unified latent space.

The causal assumption underlying this design is that, although observed EHR variables are affected by demographic heterogeneity and latent confounding, there still exists a subset of disease-relevant predictive structure that is more stable across racial groups than spurious group-dependent correlations. Accordingly, the model does not assume full causal graph identifiability from observational data alone; rather, it adopts a causal-invariance principle, namely that the most transferable predictive factors should remain relatively consistent across domains, while non-invariant factors should be absorbed into variant branches instead of dominating the final decision rule. Under this assumption, fairness is improved because the predictor is discouraged from relying excessively on demographic-specific shortcuts, and generalization is improved because the retained representation is less sensitive to source-domain-specific statistical patterns.

To enhance the stability of $F_i$, the module introduces a structured cross-sample interaction mechanism that extracts consistency across different feature channels. Given a multi-branch feature set $\{F_i^{(1)},F_i^{(2)},\dots ,F_i^{(m)}\}$, a linear transformation is applied to obtain structurally aligned representations:

15 $$\begin{aligned} u_i^{(k)}=\textrm{Linear}_k\!\left( F_i^{(k)}\right) , \qquad k=1,\dots ,m, \end{aligned}$$

where $\textrm{Linear}_k$ denotes a channel-specific linear transformation, and $u_i^{(k)}$ is the recalibrated representation for channel *k*. The module then performs element-wise fusion across channels to emphasize shared causal-invariant semantics:

16 $$\begin{aligned} \hat{u}_i = \sum _{k=1}^{m} \gamma _k \, u_i^{(k)}, \end{aligned}$$

where $\gamma _k$ are learnable channel fusion weights that quantify the contribution of each channel to the causal-invariant representation. This step further reduces the risk that the final representation is dominated by a single unstable feature branch, and instead encourages the model to retain channel components that are consistently useful across samples and domains.

Next, a Transformer Encoder is employed to capture deeper structural dependencies and obtain higher-level causal-consistent representations. The fused representation $\hat{u}_i$ is fed into the Transformer to yield the final causal-invariant feature:

17 $$\begin{aligned} z_i = \textrm{Transformer}\!\left( \hat{u}_i\right) , \end{aligned}$$

where $z_i$ denotes the high-dimensional structural causal representation derived through multi-head self-attention and feed-forward transformations. As a causal-invariant feature, $z_i$ encapsulates core information that remains stable across racial-group distribution shifts. In implementation, the Transformer encoder operates on the fused latent representation using standard self-attention and feed-forward layers, enabling the model to capture higher-order dependencies among latent components rather than relying only on shallow linear fusion.

Finally, the Causal-Invariant Domain Generalization Block feeds the causal feature $z_i$ into a linear prediction head to generate the final disease risk prediction:

18 $$\begin{aligned} \hat{y}_i = \textrm{Softmax}\!\left( W z_i + b\right) , \end{aligned}$$

where *W* and *b* are linear projection parameters, and $\hat{y}_i$ is the final predicted probability vector. Unlike standard models, this module interacts jointly with fairness-enhanced samples produced by the MedFair Diffusion Block, achieving causal-invariant prediction that is resilient to cross-group distribution shifts. During optimization, the classification objective is applied to the prediction derived from $z_i$, while the invariant feature branch is additionally regularized by the cross-domain alignment objective introduced in the training section. As a result, the learned representation is encouraged to remain discriminative for disease prediction and simultaneously consistent between source-domain and fairness-enhanced samples.

### Training objectives

To jointly improve medical fairness and cross-group domain generalization, the training objective contains two components. The first is a discriminative prediction objective defined on the labeled source-domain training samples, which ensures accurate diabetes-related risk classification. The second is an invariance objective introduced by the Causal-Invariant Domain Generalization Block, which encourages stable predictive structure in the learned representation while reducing sensitivity to demographic-specific variation. By jointly optimizing these two objectives, the model improves cross-domain robustness without overfitting to group-dependent correlations. It should be noted that the fairness-enhanced target distribution is not defined as an equal-weight mixture over group-specific empirical distributions. Instead, it is constructed only within the training split of the designated source domain as a source-side fairness-oriented augmentation distribution. The MedFair Diffusion Block approximates this distribution during generation by synthesizing fairness-enhanced samples to alleviate source-side imbalance and underrepresentation without accessing any target-domain data. The subsequent discriminative training stage does not redefine this distribution, but only regulates how real source samples and generated fairness-enhanced samples are used in downstream prediction and invariant representation learning.

To keep the generated fairness-enhanced samples compatible with the stable predictive structure of the source domain, an alignment objective is further introduced in the causal-invariant space. This objective does not force the generated samples to collapse back to the original source distribution. Instead, it aligns source-domain and fairness-enhanced samples only at the representation level, so that fairness-oriented distributional compensation is preserved while causal-invariant prediction remains stable.

19 $$\begin{aligned} \mathcal {L}_{\textrm{cls}} = - \frac{1}{N}\sum _{i=1}^{N} \sum _{c=1}^{C} y_{i,c}\log \hat{y}_{i,c} \end{aligned}$$

where *N* denotes the batch size, *C* denotes the number of classes, $y_{i,c}$ denotes the one-hot ground-truth label, and $\hat{y}_{i,c}$ denotes the predicted class probability. This loss is applied to the supervised samples used in discriminative training.

20 $$\begin{aligned} \mathcal {L}_{\textrm{inv}} = \frac{1}{N_s M}\sum _{i=1}^{N_s}\sum _{j=1}^{M} \left\| z_i^{(s)} - z_j^{(\textrm{fair})} \right\| _{2}^{2} \end{aligned}$$

where $z_i^{(s)}$ denotes the causal-invariant representation of a source-domain sample, $z_j^{(\textrm{fair})}$ denotes that of a fairness-enhanced sample, and $N_s$ and *M* denote the corresponding sample numbers. This loss constrains representation-level consistency between the two data sources, rather than directly estimating the fairness-enhanced distribution itself. Therefore, fairness-oriented sample generation at the data level and invariant alignment at the representation level work together in a complementary manner.

The overall objective is defined as follows:

21 $$\begin{aligned} \mathcal {L}_{\textrm{total}} = \mathcal {L}_{\textrm{cls}} + \lambda \mathcal {L}_{\textrm{inv}} \end{aligned}$$

where $\lambda$ is a trade-off coefficient that balances prediction accuracy and invariant representation alignment.

## Experimental results and analysis

### Evaluation metric

In the overall performance evaluation, this research first adopt classification accuracy (Accuracy) to measure the proportion of correct predictions made by the model across different demographic groups. The calculation of Accuracy is given by

22 $$\begin{aligned} \textrm{Acc}=\frac{1}{N}\sum _{i=1}^{N}\mathbb {I}\big (\hat{y}_i = y_i\big ), \end{aligned}$$

where *N* denotes the total number of samples, $y_i$ is the ground-truth label, $\hat{y}_i$ is the predicted label, and $\mathbb {I}(\cdot )$ represents the indicator function.

To further quantify the model’s discriminative ability under varying classification thresholds, this research employ the area under the receiver operating characteristic curve (AUC) as the second evaluation metric. The AUC is defined as

23 $$\begin{aligned} \textrm{AUC} =\int _{0}^{1}\textrm{TPR}(t)\,\textrm{dFPR}(t), \end{aligned}$$

where $\textrm{TPR}(t)$ and $\textrm{FPR}(t)$ denote the true positive rate and false positive rate as functions of the decision threshold.

For fairness evaluation, this research adopt the Demographic Parity Difference (DPD) to characterize disparities in positive prediction rates across demographic groups. Its mathematical definition is

24 $$\begin{aligned} \textrm{DPD} =\Big |\; \Pr (\hat{Y}=1 \mid G=a) - \Pr (\hat{Y}=1 \mid G=b) \;\Big |, \end{aligned}$$

where *G* is the demographic attribute and $\hat{Y}$ is the predicted label, measuring the overall balance of prediction outputs across groups. In the multi-racial setting of this study, DPD is computed in a pairwise manner between the source-domain group and each target-domain group under the same evaluation experiment, and the reported fairness result is obtained accordingly for each source–target transfer scenario. For binary prediction outputs, the predicted label $\hat{Y}$ is determined by thresholding the predicted probability at 0.5, that is, $\hat{Y}=1$ if the output probability is greater than or equal to 0.5, and $\hat{Y}=0$ otherwise.

Finally, this research use the Difference in Equalized Odds (DEOdds) to assess the consistency of predictions across demographic groups conditional on the true labels. The metric is defined as

25 $$\begin{aligned} \textrm{DEOdds} =\frac{1}{2}\Big ( \big |\textrm{TPR}_{a}-\textrm{TPR}_{b}\big | + \big |\textrm{FPR}_{a}-\textrm{FPR}_{b}\big | \Big ), \end{aligned}$$

where $\textrm{TPR}_{a}$, $\textrm{FPR}_{a}$ and $\textrm{TPR}_{b}$, $\textrm{FPR}_{b}$ denote the true positive and false positive rates for the two demographic groups, respectively. This metric quantifies the conditional fairness performance of the model across different populations. Similar to DPD, DEOdds is evaluated pairwise between the source-domain group and each target-domain group in each cross-domain experiment, so that fairness differences are consistently assessed under the same binary thresholding protocol. This pairwise evaluation strategy ensures that the reported fairness metrics directly reflect the disparity between the training demographic domain and the corresponding unseen evaluation demographic domain.

### Experimental setup

This study constructs cross-domain prediction and fairness evaluation scenarios based on four racial subgroups (Asian, African American, Caucasian, Hispanic), with all experiments conducted under an identical preprocessing pipeline to ensure a consistent variable space across groups. During model training, an 8:2 train–validation split is uniformly adopted, where the model is independently trained on the training split of the designated source domain. In the testing phase, out-of-domain evaluation is performed on the remaining unseen racial groups to simulate real clinical distribution shifts. Meanwhile, the MedFair Diffusion Block generates fairness-enhanced samples based only on the training data of the designated source domain and provides them to the Causal-Invariant Domain Generalization Block as additional inputs without participating in gradient updates. All experiments are conducted under the same hardware environment and hyperparameter configurations to ensure comparability across different methods and source-domain settings. To improve reproducibility, the implementation details, architectural parameters, optimization settings, diffusion configuration, and computational environment are summarized explicitly in Table 3.

**Table 3:** Summary of experimental setup.

Item	Configuration/Parameter
Data source	UCI Diabetes 130-US Hospitals Dataset
Racial groups	Asian/African American/Caucasian/Hispanic
Train–validation split ratio	8:2
Cross-domain evaluation	Train on one source-domain racial group and test on the remaining unseen racial groups
Model backbone	Encoder + CIDG Block
Fairness enhancement module	MedFair Diffusion Block (frozen during discriminative training)
Encoder hidden layers	3
Attention heads	4
Layer width	256
Transformer hidden dimension	256
CIDG projection heads	4 parallel MLP heads ($f_S$, $f_V$, $f_Y$, $f_F$), each with width 256
Activation function	ReLU
Normalization	Layer Normalization
Dropout rate	0.1
Diffusion steps	1000
Noise schedule	Linear schedule
Text-condition dimension	256
Batch size	256
Optimizer	AdamW
Learning rate	1e-4
Weight decay	1e-5
Number of epochs	100
Learning-rate scheduler	Cosine annealing
Early stopping patience	10 epochs
Framework	PyTorch
GPU	NVIDIA GeForce RTX 4090 (24 GB)
Training time	Approximately 2.47 hours per source-domain experiment
Computational setting	Single-GPU training and inference

To avoid possible target-domain leakage, all preprocessing, distribution estimation, and fairness-enhanced sample generation are conducted strictly within the training split of the designated source domain, while target-domain data are used only for out-of-domain evaluation and are never involved in model fitting, empirical distribution construction, or synthetic sample generation at any stage. Accordingly, the MedFair Diffusion Block does not access unseen target-domain samples during training, and the reported cross-domain results strictly follow the single-source-to-unseen-target domain generalization setting. In addition, all mean and standard deviation values reported in the experimental tables are computed from 5 independent runs with different random seeds, so that the reported performance reflects repeated training variability rather than a single execution result.

### Experimental results compared with other models

In the domain generalization evaluation, this research further conduct a systematic comparison between the proposed framework and several representative cross-domain generalization approaches, including FDGNet, which imposes feature perturbation and invariance constraints; Advst, which adopts an adversarial domain discriminator; DGMamba, which models distribution shift through state-space structures; MAFE, which introduces multi-view feature disentanglement; and PromptTA, which leverages prompt-based conditioning for structured transfer. By adopting a unified training and cross-domain testing protocol across the four racial subgroups, this research comprehensively examine the robustness of each model under demographic distribution disparities, clinical feature heterogeneity, and semantic drift, thereby validating the overall advantage of the proposed method in both medical fairness and cross-group predictive generalization. This research first report the results using African American as the source domain, with the remaining three racial groups serving as target domains. The corresponding experimental outcomes are presented in Table 4.

**Table 4:** Comparison of domain generalization performance using African American as the source domain. Mean ± standard deviation is reported over 5 runs.

Method	Target: Asian		Target: Caucasian		Target: Hispanic	
	Acc	AUC	Acc	AUC	Acc	AUC
FDGNet^37^	$0.597\pm 0.006$	$0.471\pm 0.008$	$0.511\pm 0.010$	$0.453\pm 0.009$	$0.560\pm 0.007$	$0.506\pm 0.006$
Advst^38^	$0.602\pm 0.009$	$0.449\pm 0.012$	$0.525\pm 0.011$	$0.447\pm 0.010$	$0.552\pm 0.008$	$0.495\pm 0.010$
DGMamba^39^	$0.611\pm 0.010$	$0.449\pm 0.009$	$0.526\pm 0.012$	$0.435\pm 0.008$	$0.545\pm 0.009$	$0.491\pm 0.011$
MAFE^40^	$0.598\pm 0.007$	$0.446\pm 0.011$	$0.503\pm 0.009$	$0.429\pm 0.012$	$0.568\pm 0.010$	$0.507\pm 0.009$
PromptTA^41^	$0.589\pm 0.008$	$0.474\pm 0.010$	$0.504\pm 0.008$	$0.431\pm 0.011$	$0.561\pm 0.007$	$0.508\pm 0.010$
**Proposed method**	$\mathbf {0.789\pm 0.005}$	$\mathbf {0.670\pm 0.006}$	$\mathbf {0.701\pm 0.006}$	$\mathbf {0.631\pm 0.007}$	$\mathbf {0.739\pm 0.004}$	$\mathbf {0.711\pm 0.006}$

The results in Table 4 show that the proposed model achieves competitive and generally stronger cross-group prediction performance than several representative domain generalization baselines when the African American group is used as the source domain and the Asian, Caucasian, and Hispanic groups are treated as target domains. The improvement is more apparent in relatively small-sample target populations, where robustness under distribution shift remains more challenging for conventional methods. In comparison, several baselines exhibit varying degrees of decline in Accuracy and AUC across target groups. A plausible reason is that the MedFair Diffusion Block enriches the source-side training distribution by introducing fairness-enhanced synthetic samples, which helps improve representation coverage for underrepresented patterns. Meanwhile, the Causal-Invariant Domain Generalization Block encourages the model to capture more stable predictive structure shared between source samples and fairness-enhanced samples, thereby improving transferability across demographic groups. Overall, these results suggest that the combination of fairness-oriented augmentation and causal-invariant representation learning is beneficial for cross-group clinical prediction.

This paper also presents experimental results using other races as source domains, as shown in Table 5.

**Table 5:** Comparison of domain generalization performance using Asian, Caucasian, and Hispanic as source domains. Mean ± standard deviation is reported over 5 runs.

Source: Asian						
Method	Target: AfricanAmerican		Target: Caucasian		Target: Hispanic	
	Acc	AUC	Acc	AUC	Acc	AUC
FDGNet	$0.248\pm 0.009$	$0.373\pm 0.011$	$0.224\pm 0.008$	$0.346\pm 0.010$	$0.286\pm 0.010$	$0.386\pm 0.009$
Advst	$0.249\pm 0.010$	$0.359\pm 0.012$	$0.244\pm 0.009$	$0.338\pm 0.011$	$0.293\pm 0.009$	$0.394\pm 0.010$
DGMamba	$0.261\pm 0.011$	$0.351\pm 0.010$	$0.233\pm 0.010$	$0.346\pm 0.009$	$0.292\pm 0.008$	$0.401\pm 0.011$
MAFE	$0.253\pm 0.010$	$0.352\pm 0.011$	$0.236\pm 0.008$	$0.352\pm 0.010$	$0.284\pm 0.009$	$0.376\pm 0.009$
PromptTA	$0.276\pm 0.009$	$0.366\pm 0.010$	$0.243\pm 0.010$	$0.356\pm 0.011$	$0.296\pm 0.008$	$0.384\pm 0.010$
**Proposed method**	$\mathbf {0.448\pm 0.008}$	$\mathbf {0.546\pm 0.010}$	$\mathbf {0.421\pm 0.009}$	$\mathbf {0.536\pm 0.010}$	$\mathbf {0.471\pm 0.007}$	$\mathbf {0.575\pm 0.009}$

Source: Caucasian						
Method	Target: Asian		Target: AfricanAmerican		Target: Hispanic	
	Acc	AUC	Acc	AUC	Acc	AUC
FDGNet	$0.231\pm 0.007$	$0.489\pm 0.012$	$0.175\pm 0.009$	$0.452\pm 0.010$	$0.226\pm 0.008$	$0.480\pm 0.011$
Advst	$0.232\pm 0.008$	$0.482\pm 0.011$	$0.192\pm 0.010$	$0.436\pm 0.012$	$0.223\pm 0.009$	$0.481\pm 0.010$
DGMamba	$0.213\pm 0.009$	$0.491\pm 0.010$	$0.178\pm 0.011$	$0.430\pm 0.009$	$0.233\pm 0.010$	$0.489\pm 0.011$
MAFE	$0.222\pm 0.009$	$0.478\pm 0.011$	$0.178\pm 0.010$	$0.436\pm 0.010$	$0.231\pm 0.009$	$0.498\pm 0.012$
PromptTA	$0.237\pm 0.009$	$0.489\pm 0.010$	$0.178\pm 0.009$	$0.431\pm 0.011$	$0.236\pm 0.010$	$0.491\pm 0.010$
**Proposed method**	$\mathbf {0.694\pm 0.006}$	$\mathbf {0.723\pm 0.008}$	$\mathbf {0.581\pm 0.008}$	$\mathbf {0.647\pm 0.009}$	$\mathbf {0.700\pm 0.007}$	$\mathbf {0.669\pm 0.008}$

**Source: Hispanic**						
Method	Target: Asian		Target: Caucasian		Target: AfricanAmerican	
	Acc	AUC	Acc	AUC	Acc	AUC
FDGNet	$0.701\pm 0.006$	$0.411\pm 0.010$	$0.666\pm 0.007$	$0.399\pm 0.009$	$0.665\pm 0.008$	$0.406\pm 0.010$
Advst	$0.684\pm 0.007$	$0.388\pm 0.011$	$0.641\pm 0.008$	$0.409\pm 0.010$	$0.639\pm 0.009$	$0.400\pm 0.011$
DGMamba	$0.689\pm 0.008$	$0.397\pm 0.010$	$0.638\pm 0.009$	$0.405\pm 0.009$	$0.658\pm 0.010$	$0.418\pm 0.012$
MAFE	$0.683\pm 0.007$	$0.408\pm 0.011$	$0.658\pm 0.008$	$0.404\pm 0.010$	$0.658\pm 0.009$	$0.414\pm 0.011$
PromptTA	$0.694\pm 0.007$	$0.402\pm 0.009$	$0.641\pm 0.008$	$0.387\pm 0.010$	$0.661\pm 0.008$	$0.408\pm 0.010$
**Proposed method**	$\mathbf {0.876\pm 0.005}$	$\mathbf {0.641\pm 0.009}$	$\mathbf {0.837\pm 0.006}$	$\mathbf {0.589\pm 0.008}$	$\mathbf {0.839\pm 0.006}$	$\mathbf {0.598\pm 0.009}$

Table 5 shows that when Asian, Caucasian, and Hispanic are respectively used as the source domain, several mainstream domain generalization methods exhibit noticeable performance variation in cross-group prediction, and this tendency appears more evident under imbalanced sample distributions. In comparison, the proposed model achieves relatively favorable Acc and AUC results across multiple cross-domain evaluation settings, while also showing comparatively smaller fluctuations in some cases, suggesting a more stable generalization trend. These results indicate that the combination of fairness-oriented augmentation and causal-invariant representation learning is helpful for alleviating performance degradation under demographic distribution shifts, thereby supporting more robust diabetes risk prediction across heterogeneous racial groups.

### Ablation test results

In order to further verify the functional roles and individual contributions of the key components in this research framework, this research conducted a systematic ablation study in which different modules were progressively incorporated to examine their stepwise performance gains. Specifically, the experiments start from a minimal Baseline model containing only the fundamental architecture, then introduce the MDB module responsible for generating fairness-enhanced samples, followed by the CIDG module designed to capture causal-invariant cross-domain representations, and finally assemble all components into the full Proposed method model. This incremental evaluation allows a clear observation of how each module independently improves cross-group prediction performance and enhances medical fairness, as well as how their combination yields complementary benefits. The corresponding ablation results are summarized in Table 6.

**Table 6:** Ablation study on the contribution of MedFair Diffusion Block (MDB) and Causal-Invariant Domain Generalization (CIDG) under different source domains. Mean ± standard deviation is reported over 5 runs.

Source: African American						
Method	Target: Asian		Target: Caucasian		Target: Hispanic	
	Acc	AUC	Acc	AUC	Acc	AUC
Baseline	$0.775\pm 0.007$	$0.644\pm 0.006$	$0.663\pm 0.008$	$0.627\pm 0.007$	$0.721\pm 0.006$	$0.685\pm 0.007$
+MDB	$0.781\pm 0.006$	$0.654\pm 0.007$	$0.678\pm 0.007$	$0.629\pm 0.006$	$0.728\pm 0.007$	$0.695\pm 0.006$
+CIDG	$0.784\pm 0.006$	$0.661\pm 0.006$	$0.688\pm 0.007$	$0.630\pm 0.006$	$0.733\pm 0.006$	$0.702\pm 0.007$
**Proposed method**	$\mathbf {0.789\pm 0.005}$	$\mathbf {0.670\pm 0.006}$	$\mathbf {0.701\pm 0.006}$	$\mathbf {0.631\pm 0.006}$	$\mathbf {0.739\pm 0.005}$	$\mathbf {0.711\pm 0.006}$

Source: Asian						
Method	Target: African American		Target: Caucasian		Target: Hispanic	
	Acc	AUC	Acc	AUC	Acc	AUC
Baseline	$0.112\pm 0.008$	$0.546\pm 0.007$	$0.113\pm 0.007$	$0.529\pm 0.007$	$0.104\pm 0.008$	$0.557\pm 0.008$
+MDB	$0.246\pm 0.009$	$0.546\pm 0.007$	$0.236\pm 0.009$	$0.532\pm 0.008$	$0.251\pm 0.009$	$0.564\pm 0.007$
+CIDG	$0.332\pm 0.008$	$0.546\pm 0.006$	$0.313\pm 0.008$	$0.534\pm 0.007$	$0.343\pm 0.008$	$0.569\pm 0.007$
**Proposed method**	$\mathbf {0.448\pm 0.007}$	$\mathbf {0.546\pm 0.006}$	$\mathbf {0.421\pm 0.007}$	$\mathbf {0.536\pm 0.006}$	$\mathbf {0.471\pm 0.006}$	$\mathbf {0.575\pm 0.006}$

Source: Caucasian						
Method	Target: Asian		Target: African American		Target: Hispanic	
	Acc	AUC	Acc	AUC	Acc	AUC
Baseline	$0.411\pm 0.008$	$0.662\pm 0.008$	$0.366\pm 0.008$	$0.628\pm 0.007$	$0.408\pm 0.008$	$0.627\pm 0.008$
+MDB	$0.524\pm 0.008$	$0.686\pm 0.007$	$0.452\pm 0.009$	$0.636\pm 0.007$	$0.525\pm 0.009$	$0.644\pm 0.007$
+CIDG	$0.595\pm 0.007$	$0.702\pm 0.007$	$0.506\pm 0.008$	$0.642\pm 0.007$	$0.598\pm 0.008$	$0.654\pm 0.007$
**Proposed method**	$\mathbf {0.694\pm 0.006}$	$\mathbf {0.723\pm 0.007}$	$\mathbf {0.581\pm 0.007}$	$\mathbf {0.647\pm 0.007}$	$\mathbf {0.700\pm 0.006}$	$\mathbf {0.669\pm 0.007}$

Source: Hispanic						
Method	Target: Asian		Target: Caucasian		Target: African American	
	Acc	AUC	Acc	AUC	Acc	AUC
Baseline	$0.526\pm 0.008$	$0.586\pm 0.008$	$0.429\pm 0.008$	$0.583\pm 0.007$	$0.462\pm 0.009$	$0.593\pm 0.008$
+MDB	$0.666\pm 0.008$	$0.608\pm 0.008$	$0.592\pm 0.008$	$0.585\pm 0.007$	$0.613\pm 0.009$	$0.595\pm 0.008$
+CIDG	$0.754\pm 0.007$	$0.622\pm 0.008$	$0.694\pm 0.007$	$0.587\pm 0.008$	$0.707\pm 0.008$	$0.596\pm 0.008$
**Proposed method**	$\mathbf {0.876\pm 0.006}$	$\mathbf {0.641\pm 0.008}$	$\mathbf {0.837\pm 0.006}$	$\mathbf {0.589\pm 0.007}$	$\mathbf {0.839\pm 0.006}$	$\mathbf {0.598\pm 0.008}$

From the overall trend, all four source-domain settings exhibit a very stable and monotonic improvement from Baseline to +MDB, +CIDG, and finally to proposed method. Whether transferring from African American to Asian, Caucasian, and Hispanic, or from Hispanic to the remaining groups, both Acc and AUC show a clear first-stage gain after incorporating the MDB module. This indicates that the MedFair Diffusion Block effectively alleviates feature-space and label-space disparities between the source and target groups, allowing the model to establish a more representative decision boundary without modifying the prediction head. The improvement is especially pronounced in scenarios with limited data or highly imbalanced label distributions, where the transition from Baseline to +MDB and subsequently to +CIDG and proposed method becomes more substantial. These observations demonstrate that the combination of generative fairness augmentation and causal-invariant modeling is particularly critical for achieving stable cross-group transfer under small-sample and long-tailed demographic conditions.

A further comparison between +MDB and +CIDG reveals their complementary roles. MDB primarily enables a shift ”from unusable to usable,” for example, lifting the extremely low Acc of Baseline to a reasonable level of cross-domain performance. On top of this, CIDG further reduces latent distribution discrepancies across demographic groups, offering more gradual and refined improvements that transform the model from merely ”capable of generalizing” to ”robustly generalizing.” Across all four source-domain configurations, the final proposed method model consistently achieves the highest Acc and AUC with lower variance, showing that the synergy between the MedFair Diffusion Block and the Causal-Invariant Domain Generalization Block not only enhances average cross-domain performance but also improves stability and reproducibility. Consequently, the proposed framework realizes stronger domain generalization and more balanced medical prediction performance across demographic groups.

**Fig. 4:**
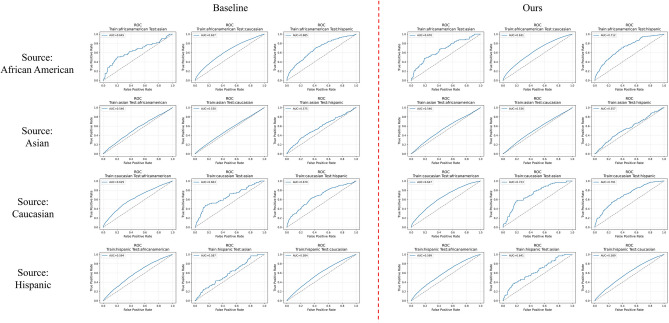
Comparison of ROC curves between the baseline and the proposed method under different source-target cross-racial evaluation settings.

Further testing is also presented here, with comparative experimental results between the proposed algorithm and the baseline, as shown in Fig. 4.

Figure 4 compares the cross-domain prediction performance of the Baseline and the proposed method through ROC curves across the four racial groups. As shown in the African American, Asian, Caucasian, and Hispanic rows, the curves produced by proposed method consistently shift toward the upper-left corner, exhibiting larger AUC values and more aligned shapes across target domains. This indicates that the proposed framework not only improves discriminative capability within each individual group but also reduces performance disparities among groups. In contrast, the Baseline curves–particularly in minority and cross-group scenarios–often lie close to the diagonal, reflecting insufficient discrimination and pronounced instability. These visual patterns directly corroborate the quantitative results presented earlier, further demonstrating that the proposed approach enhances overall predictive performance while simultaneously promoting cross-group fairness.

### Fairness analysis

Beyond performance comparison, this study further examines model behavior across different demographic groups from a fairness perspective, ensuring that overall accuracy does not obscure potential structural biases. Specifically, this research employ fairness metrics such as Demographic Parity Difference and Difference in Equalized Odds to characterize positive prediction rates and conditional error rates for the Asian, African American, Caucasian, and Hispanic groups, thereby analyzing cross-group consistency and stability at both the output distribution and decision-boundary levels. By integrating fairness evaluation with the domain generalization setting, this research assess the trade-off between ”cross-domain generalization” and ”cross-group fairness,” and verify whether the proposed framework can effectively mitigate racial disparities without substantially compromising predictive performance. The corresponding fairness evaluation results are summarized in Table 7.

**Table 7:** Fairness metrics (Demographic Parity Difference and Equalized Odds Difference across race groups) under different source domains.

Source = race_African American			Source = race_Asian		
Method	DPD (Race)	DEOdds (Race)	Method	DPD (Race)	DEOdds (Race)
Baseline	0.207	0.182	Baseline	0.231	0.205
+MDB	0.158	0.141	+MDB	0.178	0.154
+CIDG	0.112	0.096	+CIDG	0.129	0.112
**Proposed method**	**0.066**	**0.057**	**Proposed method**	**0.081**	**0.069**

Source = race_Caucasian			Source = race_Hispanic		
Method	DPD (Race)	DEOdds (Race)	Method	DPD (Race)	DEOdds (Race)
Baseline	0.219	0.191	Baseline	0.202	0.184
+MDB	0.165	0.144	+MDB	0.153	0.136
+CIDG	0.119	0.101	+CIDG	0.110	0.095
**Proposed method**	**0.073**	**0.061**	**Proposed method**	**0.067**	**0.055**

From the overall trend of fairness metrics, this research observe that regardless of which racial group is selected as the source domain, the Demographic Parity Difference (DPD) and Equalized Odds Difference (DEOdds) consistently exhibit stable and monotonic decreases as the MDB and CIDG modules are progressively introduced, ultimately reaching their lowest values in the complete proposed method model. This indicates that the fairness-enhanced samples generated by the MedFair Diffusion Block effectively mitigate systematic disparities in positive prediction rates and error rates across racial groups at the output-distribution level, thereby providing a more balanced training foundation for subsequent causal-invariant representation learning.

A further comparison among Baseline, +MDB, +CIDG, and proposed method shows that the MDB module primarily reduces prominent statistical discrepancies, making the overall decision probabilities across different groups more comparable, whereas the CIDG module, through explicit modeling of causal invariance, further compresses conditional error-rate differences and thus contributes additional improvements in DEOdds. Ultimately, the complete model achieves the lowest DPD and DEOdds values across all four source-domain settings, demonstrating its ability to suppress racial unfairness directly within the training process–without relying on post-hoc reweighting or rule-based corrections–while simultaneously preserving strong predictive performance in cross-group clinical risk prediction tasks.

### t-SNE experimental results

**Fig. 5:**
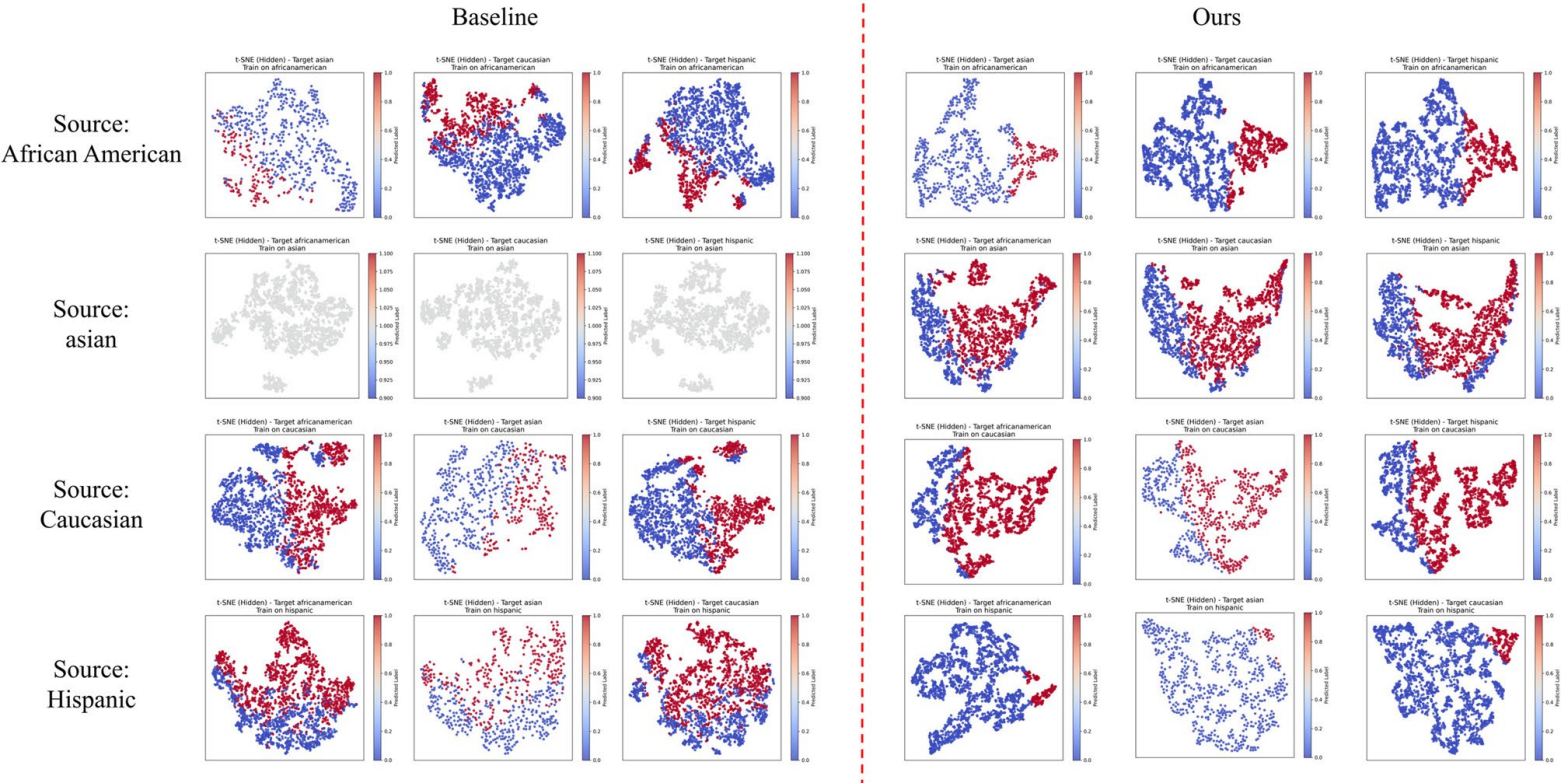
t-SNE visualization of the baseline and the proposed method under different cross-racial evaluation settings.

t-SNE visualization is employed to illustrate the structural organization of feature distributions across different domains, thereby enabling examination of the model’s separability and inter-domain aggregation patterns within the latent space. In this section, this research analyze the differences and structural characteristics of learned representations by projecting high-dimensional embeddings into a low-dimensional space. Through visual comparison, this research obtain a more intuitive understanding of the model’s representation quality and the organization of its latent space under cross-domain settings. The experimental results are shown in Fig. 5.

From the overall visualization patterns, the Baseline exhibits substantial class overlap and structural entanglement across different source-domain settings, with many samples showing unstable or weak clustering in the embedding space. This suggests that the model remains vulnerable to source-domain bias, local noise, and inconsistent statistical characteristics under cross-domain conditions. Particularly when switching between racial groups, the t-SNE projections often display large regions of interwoven or blurred sample clusters, indicating that conventional models struggle to extract domain-shared structures and fail to stably align the semantic organization of the target domain in the latent space.

In contrast, applying the proposed method results in a markedly clearer and more structured clustering pattern, where intra-class samples are more compact and inter-class boundaries become more distinct. Moreover, the global geometric structure remains consistent when projected across different source domains, demonstrating the model’s ability to effectively capture causal-invariant features, suppress domain-specific noise, and reduce bias-related variability. This leads to a more coherent and generalizable latent representation. Such improvements also align with the systematic gains observed in the generalization and fairness metrics, providing intuitive visual evidence for the effectiveness of the proposed framework.

### Confusion matrix experimental results

**Fig. 6:**
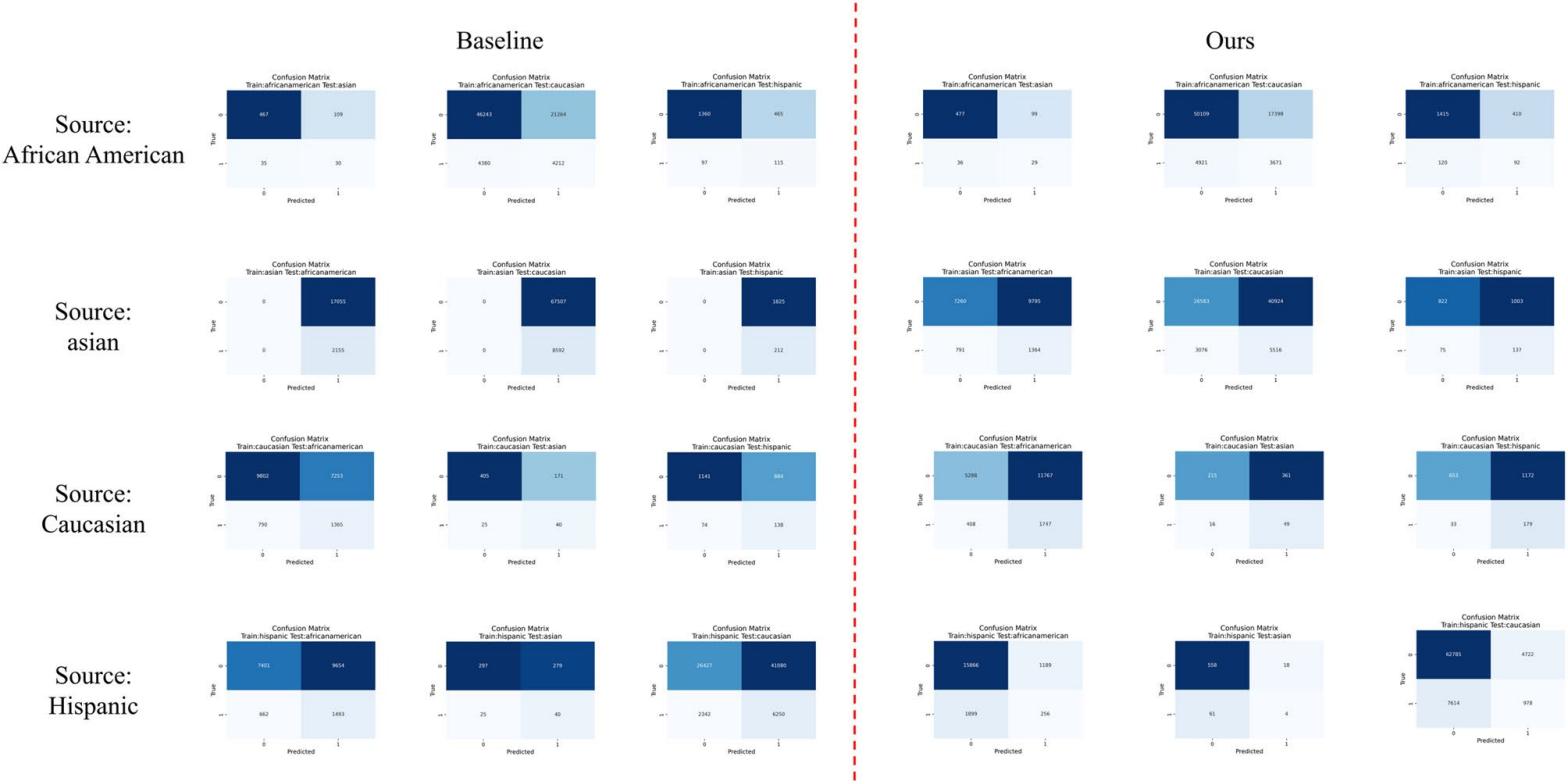
Confusion matrix comparison between the baseline and the proposed method under different cross-racial evaluation settings.

To further evaluate the model’s decision behavior across different racial domains from a fine-grained classification perspective, this section introduces confusion matrices to characterize the distribution of prediction errors and class-specific biases. Compared with relying solely on aggregate metrics, confusion matrices explicitly reveal structural differences in true positives, true negatives, and various types of misclassification, thereby exposing potential sources of bias and assessing the stability of cross-domain prediction. By comparing the prediction matrices under different source-domain configurations, this research obtain a more comprehensive understanding of the model’s behavioral patterns and classification consistency during real-world decision-making. The experimental results are presented in Fig. 6.

These confusion matrices collectively illustrate the differences in cross-domain classification behavior under various source-domain configurations. The Baseline model exhibits pronounced error concentration patterns across multiple settings, characterized by persistently elevated false-positive or false-negative regions, indicating that its decision boundaries lack robustness when confronted with heterogeneous racial distributions and are easily influenced by group-specific statistical shifts. In contrast, the proposed method demonstrates consistently more balanced distributions of true positives and true negatives across all four source domains, accompanied by significantly reduced error regions. This reflects a more stable ability to distinguish positive and negative cases while effectively suppressing cross-domain misclassification. Moreover, the overall color distribution appears more uniform, further suggesting that the proposed approach not only enhances domain generalization performance but also maintains more equitable decision behavior across different demographic groups.

### Fairness robustness under data reduction

**Fig. 7:**
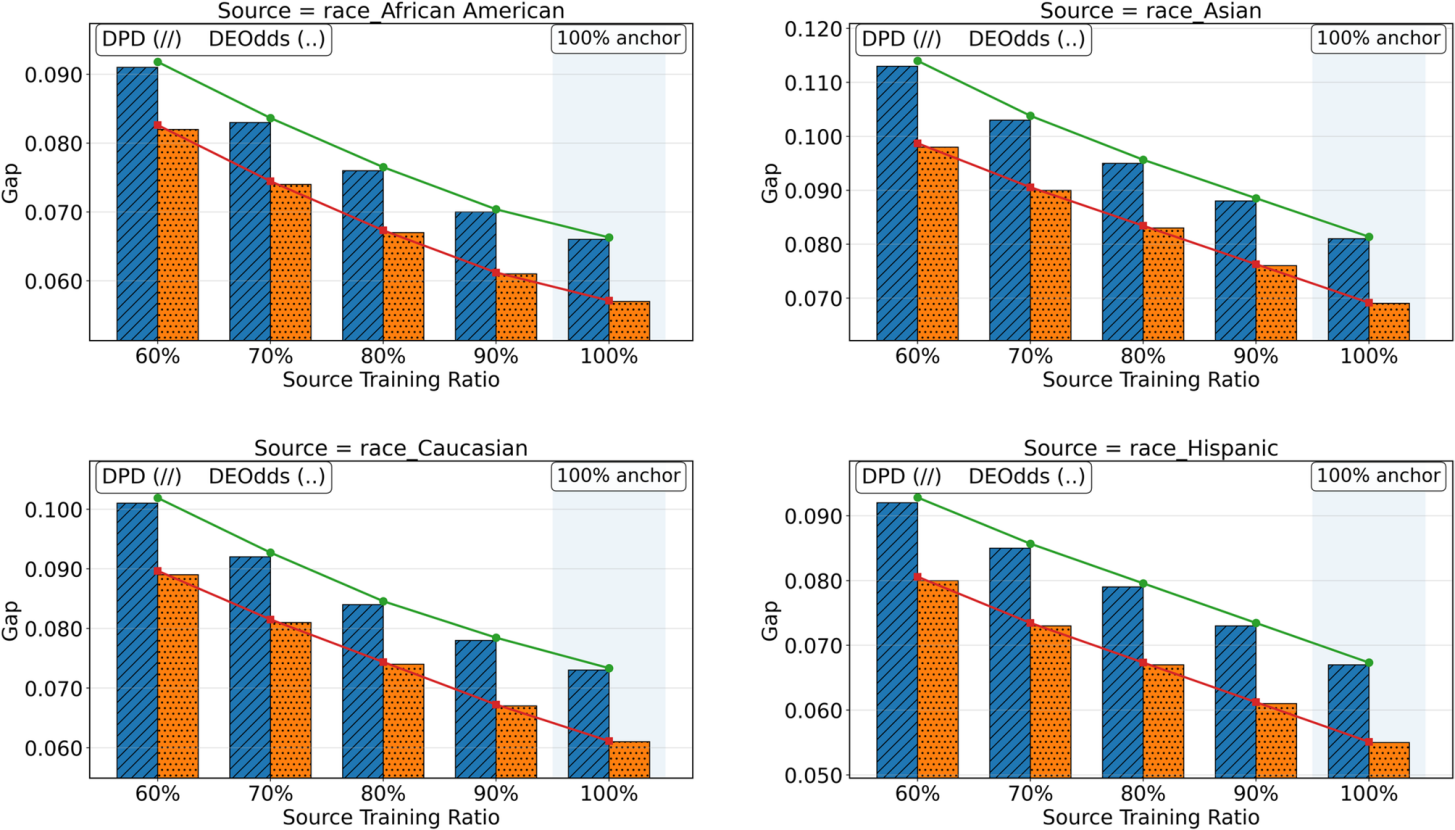
Fairness stress test under reduced source training ratios (60%–100%), showing DPD and DEOdds trends across different race source domains.

To evaluate the fairness and stability of the method under conditions of limited training data, this research designed a ”data reduction” stress test. This test simulates the uneven collection and insufficient coverage of real-world medical data by gradually reducing the proportion of available training data in the source domain. This setting allows us to observe whether the model’s ability to suppress cross-population bias remains consistent and reliable under controlled increasing difficulty as data becomes scarce. The experimental results are shown in Fig. 7.

As shown in the figure, under the four different source domain settings (African American, Asian, Caucasian, and Hispanic), as the source domain training ratio increases from 60% to 100%, both DPD and DEOdds show an overall downward trend, indicating that more sufficient source domain supervision helps to further compress cross-population prediction bias. At the same time, the descent slopes of each subgraph are not completely consistent, reflecting that the differences in data structure and population distribution of different source domains will affect the fairness convergence speed. However, the gap at the 100% anchor point remains at a low level in all settings, indicating that the method can still maintain a relatively stable bias control capability when the data is reduced, and has a certain degree of fairness robustness and transferable consistency.

### Training–inference cost trade-off

**Fig. 8:**
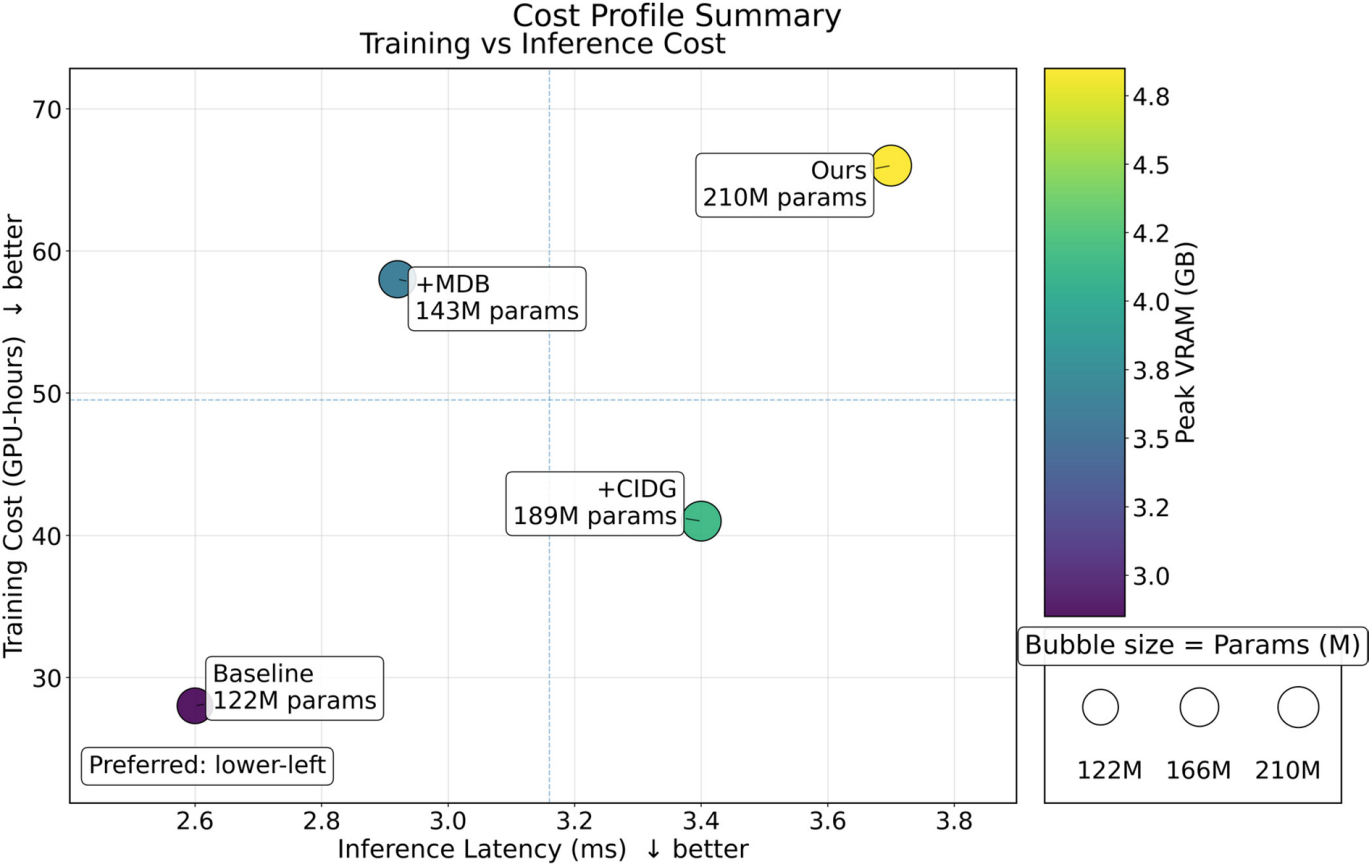
Training–inference cost comparison of different methods. Bubble size indicates parameter count, and color indicates peak VRAM usage.

To evaluate the usability and resource consumption of the method in practical deployment, this research systematically characterize the computational cost in both the training and inference phases, and uniformly quantify its time and hardware usage characteristics. This experiment focuses on inference latency and training consumption as core dimensions, while also considering model size and peak memory usage, aiming to provide a more comprehensive efficiency profile to support the engineering implementation analysis of the method. The experimental results are shown in Fig. 8.

This cost profile shows that as the method gradually introduces modules from the baseline, the overall training cost and model size increase, and the inference latency also increases to varying degrees. Although proposed method is in the upper right region (with higher training cost, latency, and memory usage), this additional cost corresponds to a more complex generation and domain generalization modeling process. In contrast, +CIDG presents a more balanced trade-off between training cost and latency, while +MDB mainly increases the burden on the training side but has a smaller impact on the inference side. Overall, it reflects the differentiated trade-offs of each module in the dimension of ”deployment efficiency - training cost”.

### Statistical robustness test

To further assess the statistical robustness of the observed improvements, this subsection reports the repeated experimental results together with their corresponding 95% confidence intervals under different source–target settings. By supplementing the mean ± standard deviation with interval estimation, the stability of the proposed method relative to the baseline can be evaluated more clearly across multiple cross-domain prediction scenarios.

**Table 8:** Comparison between Baseline and the proposed method under different source–target settings. Mean ± standard deviation is reported over 5 runs, together with the corresponding 95% confidence intervals.

Source	Target	Baseline Acc (95% CI)	Proposed method Acc (95% CI)	Baseline AUC (95% CI)	Proposed method AUC (95% CI)
African American	Asian	$$0.775\pm 0.007$$$$[0.766,\,0.784]$$	$$\mathbf {0.789\pm 0.005}$$$$\mathbf {[0.783,\,0.795]}$$	$$0.644\pm 0.006$$$$[0.637,\,0.651]$$	$$\mathbf {0.670\pm 0.006}$$$$\mathbf {[0.663,\,0.677]}$$
African American	Caucasian	$$0.663\pm 0.008$$$$[0.653,\,0.673]$$	$$\mathbf {0.701\pm 0.006}$$$$\mathbf {[0.694,\,0.708]}$$	$$0.627\pm 0.007$$$$[0.618,\,0.636]$$	$$\mathbf {0.631\pm 0.006}$$$$\mathbf {[0.624,\,0.638]}$$
African American	Hispanic	$$0.721\pm 0.006$$$$[0.714,\,0.728]$$	$$\mathbf {0.739\pm 0.005}$$$$\mathbf {[0.733,\,0.745]}$$	$$0.685\pm 0.007$$$$[0.676,\,0.694]$$	$$\mathbf {0.711\pm 0.006}$$$$\mathbf {[0.704,\,0.718]}$$
Asian	African American	$$0.112\pm 0.008$$$$[0.102,\,0.122]$$	$$\mathbf {0.448\pm 0.007}$$$$\mathbf {[0.439,\,0.457]}$$	$$0.546\pm 0.007$$$$[0.537,\,0.555]$$	$$\mathbf {0.546\pm 0.006}$$$$\mathbf {[0.539,\,0.553]}$$
Asian	Caucasian	$$0.113\pm 0.007$$$$[0.104,\,0.122]$$	$$\mathbf {0.421\pm 0.007}$$$$\mathbf {[0.412,\,0.430]}$$	$$0.529\pm 0.007$$$$[0.520,\,0.538]$$	$$\mathbf {0.536\pm 0.006}$$$$\mathbf {[0.529,\,0.543]}$$
Asian	Hispanic	$$0.104\pm 0.008$$$$[0.094,\,0.114]$$	$$\mathbf {0.471\pm 0.006}$$$$\mathbf {[0.464,\,0.478]}$$	$$0.557\pm 0.008$$$$[0.547,\,0.567]$$	$$\mathbf {0.575\pm 0.006}$$$$\mathbf {[0.568,\,0.582]}$$
Caucasian	Asian	$$0.411\pm 0.008$$$$[0.401,\,0.421]$$	$$\mathbf {0.694\pm 0.006}$$$$\mathbf {[0.687,\,0.701]}$$	$$0.662\pm 0.008$$$$[0.652,\,0.672]$$	$$\mathbf {0.723\pm 0.007}$$$$\mathbf {[0.714,\,0.732]}$$
Caucasian	African American	$$0.366\pm 0.008$$$$[0.356,\,0.376]$$	$$\mathbf {0.581\pm 0.007}$$$$\mathbf {[0.572,\,0.590]}$$	$$0.628\pm 0.007$$$$[0.619,\,0.637]$$	$$\mathbf {0.647\pm 0.007}$$$$\mathbf {[0.638,\,0.656]}$$
Caucasian	Hispanic	$$0.408\pm 0.008$$$$[0.398,\,0.418]$$	$$\mathbf {0.700\pm 0.006}$$$$\mathbf {[0.693,\,0.707]}$$	$$0.627\pm 0.008$$$$[0.617,\,0.637]$$	$$\mathbf {0.669\pm 0.007}$$$$\mathbf {[0.660,\,0.678]}$$
Hispanic	Asian	$$0.526\pm 0.008$$$$[0.516,\,0.536]$$	$$\mathbf {0.876\pm 0.006}$$$$\mathbf {[0.869,\,0.883]}$$	$$0.586\pm 0.008$$$$[0.576,\,0.596]$$	$$\mathbf {0.641\pm 0.008}$$$$\mathbf {[0.631,\,0.651]}$$
Hispanic	Caucasian	$$0.429\pm 0.008$$$$[0.419,\,0.439]$$	$$\mathbf {0.837\pm 0.006}$$$$\mathbf {[0.830,\,0.844]}$$	$$0.583\pm 0.007$$$$[0.574,\,0.592]$$	$$\mathbf {0.589\pm 0.007}$$$$\mathbf {[0.580,\,0.598]}$$
Hispanic	African American	$$0.462\pm 0.009$$$$[0.451,\,0.473]$$	$$\mathbf {0.839\pm 0.006}$$$$\mathbf {[0.832,\,0.846]}$$	$$0.593\pm 0.008$$$$[0.583,\,0.603]$$	$$\mathbf {0.598\pm 0.008}$$$$\mathbf {[0.588,\,0.608]}$$

Table 8 shows that the proposed method maintains consistently narrower or clearly shifted 95% confidence intervals with higher mean performance than the baseline in most source–target settings, indicating that the observed gains are not caused by isolated fluctuations in repeated runs. The improvement is particularly evident for Acc, where the interval ranges of the proposed method are generally well above those of the baseline across multiple cross-domain transfer tasks, while the AUC gains are also stable in several settings, especially under African American, Caucasian, and part of Hispanic source domains. Although the AUC improvement is relatively modest in a few challenging transfer cases, the interval estimates still suggest that the overall trend of the proposed framework remains more robust and reliable than that of the baseline under cross-group distribution shifts.

## Discussion and limitations

Although the fairness evaluation in this study focuses primarily on racial groups, this choice is mainly constrained by the characteristics of the adopted dataset. In the Diabetes 130-US hospitals dataset, race is one of the relatively explicit and consistently recorded demographic attributes, whereas other potentially important protected attributes, such as socioeconomic status, insurance-related indicators, education level, or more fine-grained social determinants of health, are either unavailable, indirectly represented, or subject to substantial missingness and limited reliability. Although variables such as gender and age are available, constructing a unified multi-attribute fairness evaluation framework under cross-domain settings would require more complete subgroup annotations and sufficient sample support within each intersecting demographic category to ensure statistically meaningful comparison. In addition, publicly available healthcare datasets that simultaneously provide structured EHR variables, reliable clinical outcome labels, and sufficiently complete demographic annotations for cross-racial fairness and domain generalization evaluation remain very limited. For this reason, the present study uses the Diabetes 130-US hospitals dataset as a representative benchmark for method validation under the current experimental scope. Therefore, the current study concentrates on race as the primary fairness dimension under the present data conditions. Nevertheless, the proposed framework is not inherently restricted to a single protected attribute. If richer and more complete demographic annotations are available, the diffusion-based fairness augmentation mechanism and the causal-invariant representation learning strategy can be naturally extended to incorporate multiple protected attributes and their joint interactions, which remains an important direction for future work.

From a practical perspective, the fairness-enhanced synthetic samples are introduced only during model training to compensate for minority-group underrepresentation and to encourage the predictor to learn a more balanced and stable decision boundary; they are not used as direct substitutes for real patient records in downstream clinical deployment or individual-level medical decision-making. Therefore, their role is to improve model calibration, fairness, and cross-group robustness at the algorithmic level rather than to generate clinically actionable patient profiles. Nevertheless, the use of synthetic data in medical AI still requires careful ethical and practical consideration, because unrealistic or distribution-distorted generated samples may introduce hidden bias amplification, misleading feature associations, or reduced clinical interpretability if not properly controlled. In addition, although the observed improvements in predictive performance and fairness metrics indicate the potential value of the proposed framework, these gains should be interpreted with appropriate caution in real-world settings, where deployment conditions are more complex than controlled experimental environments. Future work will further evaluate the proposed framework on data collected from different hospitals to examine its robustness under broader clinical environments and institutional heterogeneity. Such multi-center validation will help provide stronger empirical support for the real-world generalizability and practical applicability of the proposed method.

## Conclusion

This study proposes a unified framework that integrates diffusion-based generation with causal-invariant modeling to simultaneously enhance predictive accuracy and decision fairness in multi-racial diabetes risk prediction. By constructing the MedFair Diffusion Block, the method performs mixture-based modeling and conditional generation over the empirical distributions of different racial subgroups, thereby mitigating the bias risks introduced by sample imbalance and distributional disparities at the data level. At the same time, the framework incorporates a Causal-Invariant Domain Generalization module, which explicitly decomposes stable semantics, cross-group varying factors, and fairness-related features within a shared latent space, and aligns the structural representations of source-domain and fairness-enhanced samples through invariance constraints. Systematic experiments on publicly available multi-racial diabetes datasets demonstrate that under cross-racial transfer settings, the proposed approach not only maintains or significantly improves accuracy and AUC, but also effectively reduces fairness metrics such as Demographic Parity Difference and Equalized Odds Difference, validating its practical utility and robustness in heterogeneous clinical environments.

Looking ahead, several research directions remain to be explored in order to address the more complex population structures and clinical pathways observed in real-world medical settings. On one hand, the MedFair risk modeling paradigm may be extended to temporal disease progression prediction and multi-task learning scenarios, enabling unified fairness-enhanced modeling for a range of clinical endpoints such as medication response, complication onset, and readmission risk. On the other hand, integrating causal-invariant modeling with more fine-grained social determinants of health and institution-level variables may help characterize the indirect impacts of policy, resource allocation, and environmental factors on model decisions. Furthermore, future research may incorporate multimodal medical data–including imaging, clinical text, and genomic information–to evaluate and constrain fairness across population groups at higher representational levels, ultimatel

## Data Availability

The data used in this study are publicly available from the UCI Machine Learning Repository as the ”Diabetes 130-US hospitals for years 1999–2008” dataset. It can be accessed and downloaded from: https://archive.ics.uci.edu/dataset/296/diabetes+130+us+hospitals+for+years+1999+2008. This work analyzes derived subsets constructed by stratifying the original dataset according to the race attribute (Asian, African American, Caucasian, and Hispanic) and applying a unified preprocessing pipeline, including missing-value imputation, outlier trimming, categorical re-encoding with consistent mappings, and numerical feature standardization.
